# Integrin-linked kinase (ILK): the known vs. the unknown and perspectives

**DOI:** 10.1007/s00018-021-04104-1

**Published:** 2022-01-28

**Authors:** Agata Górska, Antonina Joanna Mazur

**Affiliations:** Department of Cell Pathology, Faculty of Biotechnology, University of Wroclaw, ul. Joliot-Curie 14a, 50-383 Wrocław, Poland

**Keywords:** Cancer, Development, Multidrug resistance (MDR), Epithelial-mesenchymal transition (EMT), Integrin-linked kinase (ILK), Cell adhesion, Migration, Signaling, Exosomes/extracellular vesicles

## Abstract

Integrin-linked kinase (ILK) is a multifunctional molecular actor in cell–matrix interactions, cell adhesion, and anchorage-dependent cell growth. It combines functions of a signal transductor and a scaffold protein through its interaction with integrins, then facilitating further protein recruitment within the ILK–PINCH–Parvin complex. ILK is involved in crucial cellular processes including proliferation, survival, differentiation, migration, invasion, and angiogenesis, which reflects on systemic changes in the kidney, heart, muscle, skin, and vascular system, also during the embryonal development. Dysfunction of ILK underlies the pathogenesis of various diseases, including the pro-oncogenic activity in tumorigenesis. ILK localizes mostly to the cell membrane and remains an important component of focal adhesion. We do know much about ILK but a lot still remains either uncovered or unclear. Although it was initially classified as a serine/threonine-protein kinase, its catalytical activity is now questioned due to structural and functional issues, leaving the exact molecular mechanism of signal transduction by ILK unsolved. While it is known that the three isoforms of ILK vary in length, the presence of crucial domains, and modification sites, most of the research tends to focus on the main isoform of this protein while the issue of functional differences of ILK2 and ILK3 still awaits clarification. The activity of ILK is regulated on the transcriptional, protein, and post-transcriptional levels. The crucial role of phosphorylation and ubiquitylation has been investigated, but the functions of the vast majority of modifications are still unknown. In the light of all those open issues, here we present an extensive literature survey covering a wide spectrum of latest findings as well as a past-to-present view on controversies regarding ILK, finishing with pointing out some open questions to be resolved by further research.

## Introduction

Following its discovery in 1996 by Hannigan and colleagues, Integrin-linked kinase (ILK) emerged as a receptor–proximal protein kinase and a new molecular actor in cell adhesion and anchorage-dependent cell growth [1]. Although ILK was initially described as a serine/threonine–protein kinase, there had still been considerable controversy surrounding the issue of the exact molecular mechanism of signal transduction by ILK [2] until it was proven that ILK is a pseudokinase [3]. It has been primarily detected at focal adhesion (FA) sites [4], confirmed by co-localization with focal adhesion markers like vinculin and paxillin [5]. Subsequently, association with its specific binding partners, such as particularly interesting cys-his-rich protein (PINCH) [4] and β-Parvin [6, 7] within FAs has been revealed. ILK combines functions of a signal transductor and a scaffold protein by interacting with the cytoplasmic domains of the β1 and β3 subunits of integrin receptors, then facilitating further protein recruitment within the ILK–PINCH–Parvin complex (IPP) [8–11]. The IPP complex orchestrates bidirectional signaling between the extracellular matrix (ECM) and intracellular compartments [8]. Thus, it takes part in the regulation of cells’ shape, migration, survival, differentiation, and gene expression. Involvement in those processes, coupled with its crucial role in epithelial–mesenchymal transition (EMT), invasion, and angiogenesis, indicated ILK as an attractive target for tumor treatment. Essentially, upregulation of *ILK* expression has been reported in human malignancies, being associated with poor prognosis of patients’ survival; thus, emphasizing its role in cancer diagnosis and prognosis. In physiological conditions, ILK is also involved in developmental processes both on the cellular [12] and embryonic level [13–16]. Going further, the implication of ILK in aging processes has been suggested as well [17]. Also, the crucial role of ILK in the cardiovascular system has been reported, particularly in processes of neovascularization and cardiomyogenesis [18–20], which is additionally supported by the fact that mutations in the *ILK* gene may be linked with cardiomyopathy in humans [21, 22]. Knockout experiments on *Caenorhabditis elegans*, *Drosophila melanogaster*, *Xenopus laevis*, and *Mus musculus* have revealed embryonic lethality linked to adhesive and migratory defects [13, 15, 23, 24]. All those functions of ILK are controlled through a myriad of signaling pathways including phosphoinositide 3-kinases/protein kinase B (PI3K/Akt) [25], mammalian target of rapamycin (mTOR) [26], nuclear factor-kappa B (NF-κB) [27, 28], glycogen synthase kinase 3-beta (GSK3-β) [29–31], cell division control protein 42 homolog (Rac/Cdc42) [32–36], snail1/E-cadherin [37–39], and others [11, 40, 41].

Although ILK has received much attention over the last two decades, some areas of our knowledge, for example, its isoforms, subcellular localization, nuclear shuttling, have been overlooked in previous reviews, while views on other issues—such as structure and presumed kinase activity have been dynamically evolving throughout the time. Variety of contradictory findings and opinions leave some crucial pieces of our knowledge controversial or yet unestablished, creating space for hypotheses still awaiting further clarification in upcoming research. The first step to a possible breakthrough would be an unbiased summary of cross-sectional knowledge and past-to-present beliefs. Responding to these needs, our review sheds new light on integrin-linked kinase in an extensive literature survey covering a broad spectrum of latest findings, including references to already released reviews, then points out some open questions to be resolved by further research**.** We want to stress that we cover only briefly areas, on which there are splendid reviews, which we list in this review.

## Genomic localization, transcripts, and isoforms of ILK

**Fig. 1:**
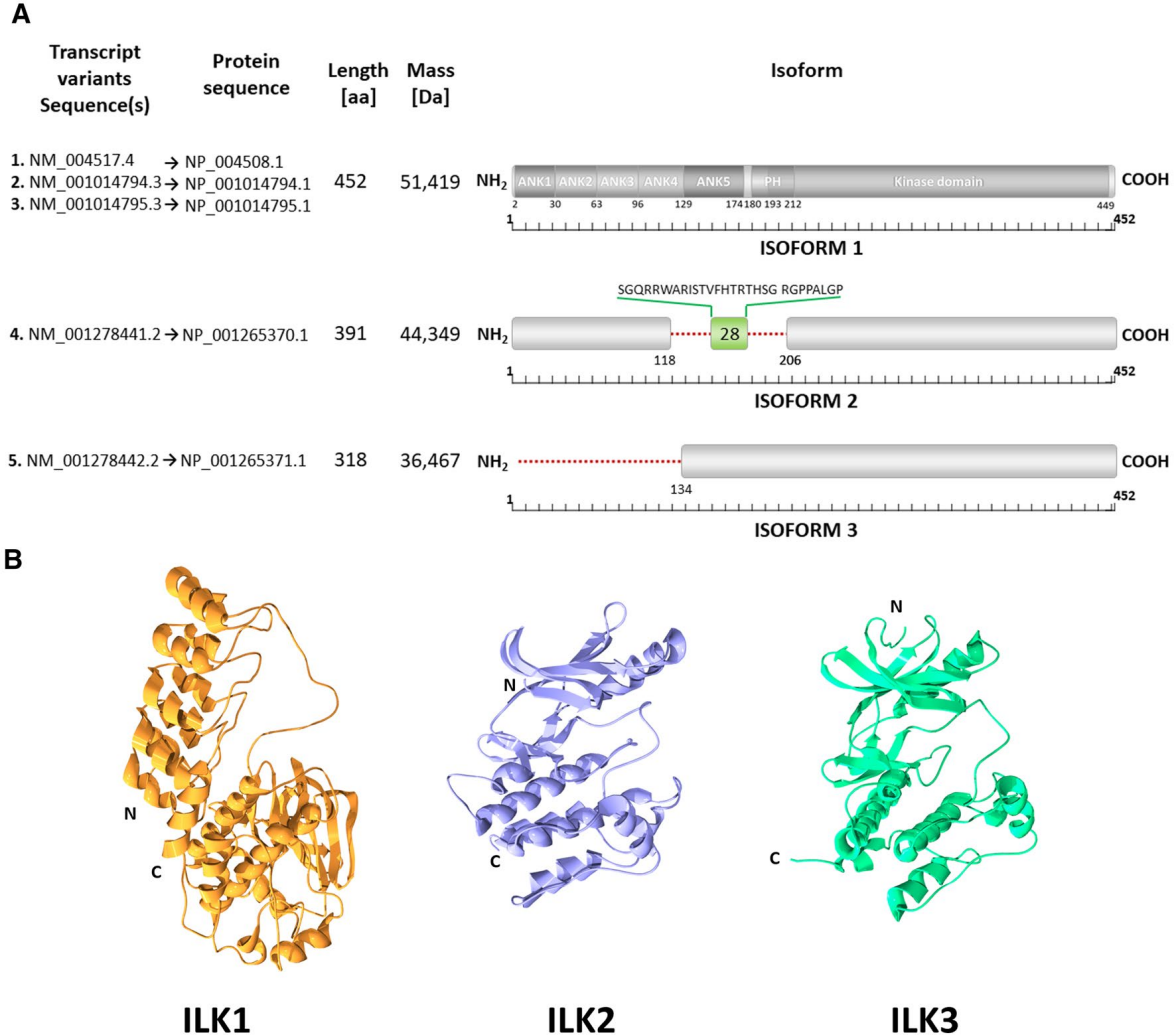
Schematic representation of ILK isoforms (**A**) and cartoon prediction of their quaternary structures (**B**). **A** This figure was prepared based on data available elsewhere [44, 47]. Accession numbers are referenced to Gene and Protein sections of the NCBI database. UniProt entries are as follows: ILK1—Q13418-1, ILK2—Q13418-2 and ILK3—Q13418-3. Sequences of isoforms 2 and 3 are compared to canonical isoform 1. The red dotted line indicates missing fragments of the sequence, while the green box inserted sequence. For the sake of orientation which domains are missing in ILK2 and ILK3 on the schematic representation of ILK1, there are marked following domains: ANK—Ankyrin repeat, PH—pleckstrin homology-like domain, and kinase domain (KD). **B** Cartoon representation of predicted quaternary structures of ILK isoforms. Predicted 3-D projections of full-length ILK1, ILK2, and ILK3. ILK1 representation was based on PDB entries #3REP and #4HI9. ILK2 representation is based on PDB entry #3REP.1.A., while ILK3 representation is based on PDB entry #6mib.1. Predictions were prepared based on the above structures, which were next merged and modified in Swiss-PdbViewer (aka DeepView) [48] desktop with ILKs alpha-model generated in Swiss Model online tool software, and finally rendered in POV-Ray 3.7.0 (Persistence of Vision Raytracer Pty. Ltd)

**Fig. 2:**
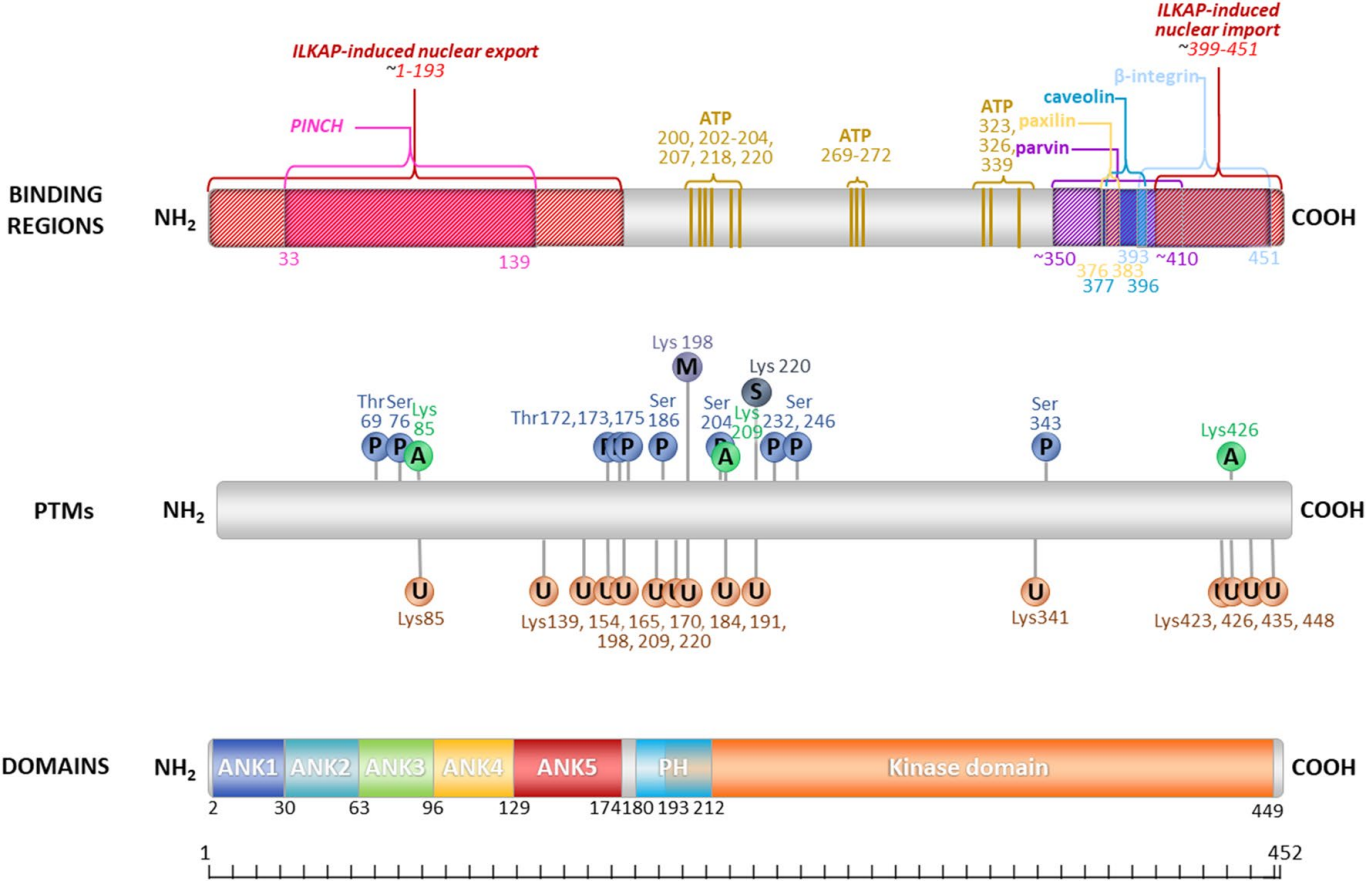
Modular distribution of functional regions, post-translational modifications (PTMs) and domains of integrin-linked kinase. Only the regions to which ILK’s most critical molecular partners bind are marked here. For exact sources (references) of the data on domain localization, see the text of chapter 4. Phosphorylation sites were taken from PhosphoSitePlus, a database of mammalian post-translational modifications (PTMs), including phosphor (P)-, acetyl (A)- and methyl (M)-groups as well as Ubiquitin (U) and SUMO (S) modifications. Over 95% of the sites were confirmed by Mass Spectrometry (MS) experiments. Early MS data have been reanalyzed for data reliability improvement, applying a common standard of analysis across over 1 000 000 spectra [49, 50]. ATP binding sites were taken from [51]

**Fig. 3:**
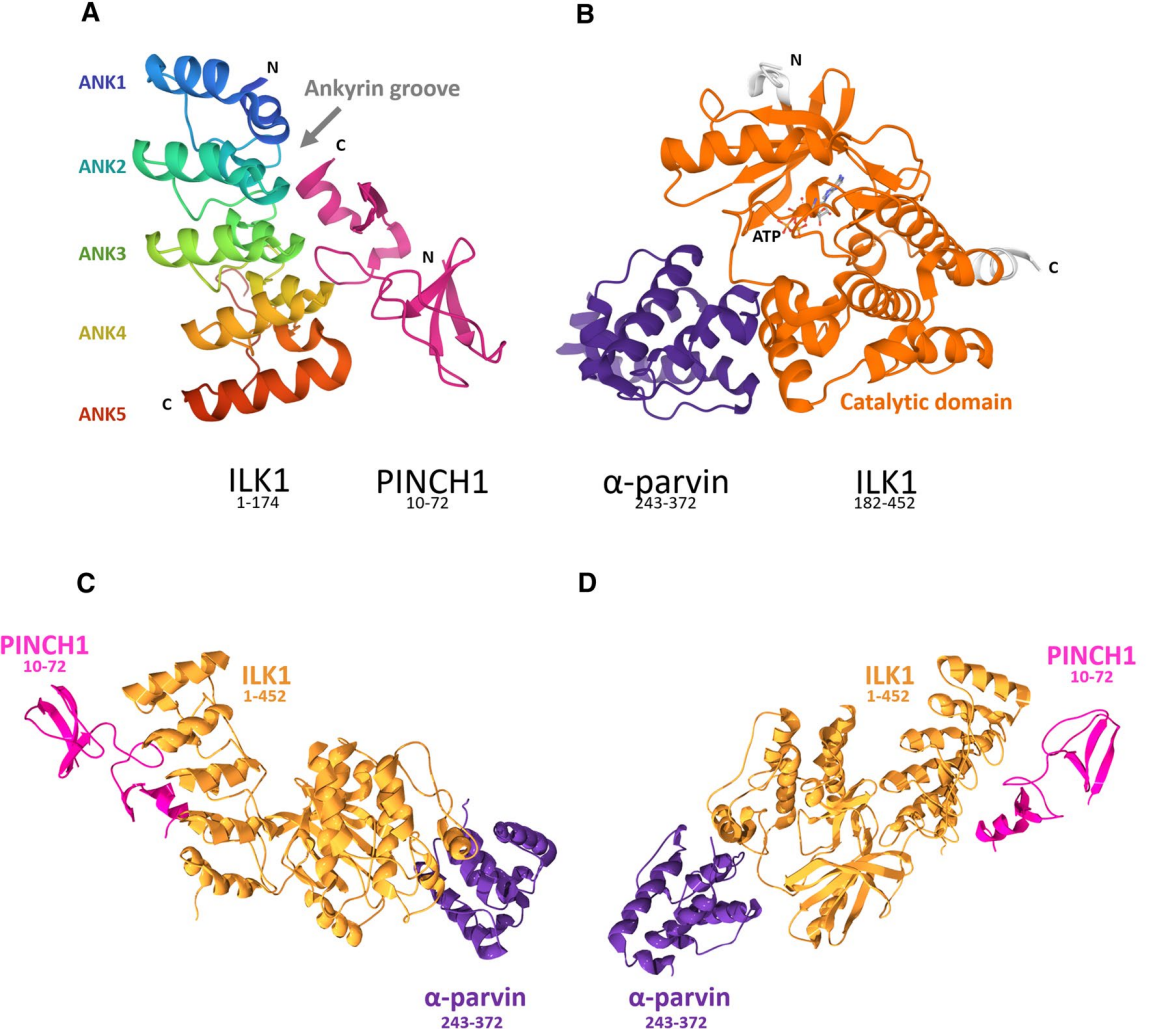
Cartoon representation of quaternary structures of ILK:PINCH1, ILK:α-Parvin and IPP complexes. **A** ILKs ankyrin repeats region (residues 1–174) in complex with PINCH1 (residues 10–72). Resolution: 1.2 Å; PDB #4HI9. **B** Region containing the catalytic domain of ILK (residues 182–454) in complex with α-Parvin (residues 243–372). Resolution: 1.8 Å; PDB #3REP. **C** and **D** Predicted 3-D projections of full-length ILK with its ligands. Predictions were prepared based on the above structures, which were as next merged and modified in Swiss-PdbViewer (aka DeepView) [48] desktop with ILKs alpha-model generated in Swiss Model online tool software, and finally rendered in POV-Ray 3.7.0 (Persistence of Vision Raytracer Pty. Ltd)

The chromosomal position of the gene encoding ILK has been localized to 11p15.5-p15.4 [42]. There are five transcription variants of this gene, encoding three isoforms (Fig. 1). Variants 1, 2, and 3 encode the same isoform 1, namely ILK1 (51 kDa)—most widely described in the literature, thus selected as canonical. Variant 1 is the primary one, while two other variants differ in coding or processing the 5’UTR regions [43, 44]. Two remaining isoforms are shorter than the canonical sequence of ILK1, each encoded by one corresponding transcript. Hence, variant 4, lacking two alternate exons in the coding region, causing a frameshift, is translated as Isoform 2 (44 kDa). As a result, the ILK2 protein lacks a fragment covering a domain resembling the pleckstrin homology (PH) domain, as well as part of the ankyrin repeat domain (ARD), including the Threonine 173 phosphorylation site (for graphic representation of the structural composition of ILK see Fig. 2). Instead of this fragment, 28 different amino acids are inserted. Finally, Variant 5, lacking an exon within the 5′ coding region, undergoes alternative splicing in the 5′ UTR, and its translation is initiated at a downstream in-frame start codon, producing isoform 3 (36 kDa), which has shorter N-terminus, as compared to isoform 1 [44–46]. According to the UniProt database, all three isoforms exist on a protein level. Taking advantage of resolved structures of N- and C-terminal parts of ILK and using appropriate software, we generated complete structural representations of ILK1 and the other two ILK isoforms (Fig. 1B). In the case of ILK2 and ILK3, the ARD disappears, suggesting that these isoforms could not bind PINCH1 (for the binding interface of ILK:PINCH1 complex, please see Fig. 3A, C, D). Moreover, the kinase domain (KD) seems to be reorganized as well; thus, ILKs interaction with α-Parvin could also be distorted (for the binding interface of ILK:α-Parvin complex, please see Fig. 3B–D).

Despite the awareness of the presence of ILK isoforms, most studies tend to focus on ILK1, failing to distinguish the unique characteristics of others. That might result from the specifity of used antibodies. For instance, antibodies directed against N-terminus of ILK1 will not detect ILK3. The same situation might be valid for ILK2 while using antibodies recognizing the central part of the ILK moiety. For example, one of the antibodies against ILK (HPA048437, Merck) is directed against residues from 118 to 241 of ILK1, which overlap with the long fragment of ILK3 (107 out of 124 residues, 86% of the immunogen) and barely 37 residues of ILK2 (29% of the immunogen). Henceforward, those antibodies shall also recognize ILK3 and probably have no or low affinity to ILK2.

To the best of our knowledge, only one research article addressed the issue of different functional characteristics of ILK1 and ILK2. Level of ILK2 occurred to be regulated in a TGF-β1 (Transforming Growth Factor β1)-dependent manner, exclusively in a highly invasive melanoma cell line but not in normal adult tissues [52, 53]. On the other hand, it was noted that ILK1 is ubiquitously expressed in normal tissues, but also upregulated in various malignancies, independently of TGF-β1 stimulation [43]. Concluding from the structural differences within essential domains described for ILKs isoforms, their functional properties are likely to vary. Considering the complexity of ILKs interactome, taken together with the current gap in knowledge on the role of particular isoforms in those processes, further exploration of the functional differences between those is one of the most intriguing issues for further research.

Despite some recent controversy on the role of alternative splicing [54], past-to-present literature provides a plethora of evidence of differential functions of proteins’ splice variants or isoforms, mainly investigated in the matter of cellular pathology, including carcinogenesis (for details, see general reviews [55, 56] or those written on examples of specific proteins [57–59]), but in nonpathological conditions as well (for instance gelsolin, addressed in [60, 61]). Thus, studies on different ILK isoforms might bring new exciting data. Due to the lack of recognition between the ILK isoforms in most of the existing studies, if not stated otherwise, henceforth, it will be spoken exclusively of ILK1.

## Regulation of ILK level and activity

### Regulation of *ILK* expression level

The *ILK* gene shares some characteristic features of housekeeping genes such as TATA-less and GC-rich promoter region with plenty of motifs capable of binding transcription factors, such as AP-2, Sp1, and NF-κβ [43]. Still, the exact characteristics of transcription factors regulating *ILK* expression are far from understanding. However, an increasing number of studies have proven that regulation of *ILK* expression depends on Sp1 protein level and Sp1 DNA-binding activity in the *ILK* promoter [62, 63]. Conversely, increased expression of AP-2α is correlated with the downregulation of *ILK* expression [64]. Furthermore, Sp1 and AP-2α have been demonstrated to act synergistically in the regulation of *ILK* promoter [64]. Integrin αVβ3 might be associated with positive regulation of promoter activity either through Sp1 [63] or involving the binding of Ets-1 to the *ILKs* second Ets DNA motif [65, 66]. Lastly, hypoxic conditions have been proven to upregulate *ILK* expression through HIF-α action [67, 68]. In summary, various transcription factors can regulate *ILK* expression. Further deciphering their interplay would be relevant, especially in the context of malignancies.

### Regulation of mRNA level by miRNAs

The ILK gene expression has also been proved to be suppressed by miRNAs through binding to ILKs mRNA 3′-UTR. Such inhibition has been confirmed for miR-542-3p in oral squamous cell carcinoma cells [69] and gastric adenocarcinoma cells [70]. Similar repression of *ILK* expression has been described through miR-625, which was linked to ameliorating lymphatic metastasis in human gastric cancer cells both in vitro and in vivo [71]. Another report indicates that *ILK* migt be downregulated by synergistic interaction of miR-145 and miR-143, projecting on inhibiting growth of bladder cancer cells [72].

### Control of ILKs level and its activity by post-transcriptional modifications (PTMs)

High-throughput mass spectrometry data reported several dozen post-translational modifications (PTMs) of ILK, including phosphorylation, acetylation, ubiquitylation, SUMOylation, and methylation. However, their exact functions are predominantly yet to be clarified [49, 50]. Many phosphorylation sites have been in focus of the proteomic as well functional research, among which Serine 246, Threonine 173, Threonine 181, Serine 259, and Serine 343 have been gaining the most attention. Further progress on this topic might be expected since dedicated antibodies for detecting some of those sites are currently available. However, experimental verification of those antibodies is still awaited. To date, there is just one approach in the literature validating their application, although not on appropriately mutated versions of ILK [73]. Early reports have suggested Serine 343 of ILK to be the target of autophosphorylation [74]. It was further evaluated that this amino acid residue is essential for following phosphorylation of ILKs downstream targets and its putative interaction with PKB/Akt [25, 74]. The majority of physiological processes regulated by ILK have been found to overlap with those controlled by p21-activated kinase 1 (PAK1), suggesting a crucial role of ILKs phosphorylation by PAK1 in its functions [75]. An essential role in the negative regulation of ILKs activity is played by phosphatases, such as ILK-associated protein (ILKAP) [76] or phosphatase and tensin homolog deleted on chromosome 10 (PTEN) [77]. Together with Chromosomal Maintenance 1 (CRM1), also known as Exportin 1, ILKAP is also implicated in the nucleo-cytoplasmic shuttling of ILK, facilitating its export to the cytoplasm. Studies suggest that ILK is imported into the nucleus through the N-terminal nuclear localization sequence (Fig. 2) via active transport mechanisms involving nuclear pore complexes [69]. Mapping studies of the ILK sequence suggest that the first 192 N-terminal residues are crucial for induction of the nuclear localization. At the same time, the 52 amino acid long fragment on its C-terminus is pivotal in triggering ILKAP-induced nuclear export [69, 75]. Going further, the nuclear export of ILK is also dependent on phosphorylation of its Threonine 173 and Serine 246 residues by PAK1, which was confirmed both in vitro and in vivo [75]. Investigation of pathways involved in axon guidance revealed that phosphorylation of Threonine 173 and Threonine 181 occurs through protein kinase Cα (PKCα) [70].

Interestingly, ubiquitylation of ILK has been reported to be involved in the regulation of its degradation, both through the endocytic–lysosomal pathway and proteasomal pathway. Indeed, to our best knowledge, there are 14 known ubiquitylation sites of ILK [49, 50] (Fig. 2), but the current literature lacks evaluation of exact functions for each of them. An essential role in those processes is played by Hsp90, which is not only required for formation and stability of ILK complexes, but also is crucial for the regulation of ILKs degradation pathways. Inhibition of Hsp90 promotes ILK ubiquitylation by the E3 ubiquitin ligase C terminus of HSC70-interacting protein (CHIP), which triggers the degradation of ILK in a proteasome [71]. Furthermore, another report suggests that ubiquitylation of ILK preceded specifically by induction by nitric oxide might lead alternatively to the degradation of ILK exclusively through the endocytic–lysosomal pathway [72].

Taken together, the current knowledge on the multilevel regulation of ILK leaves much to explore in further research. New protein–protein interactions are awaiting discovery, and potential crosstalk between each level of regulation shall be taken into account in a broader perspective. That may eventually lead to the clarification of ILK’s exact mechanisms of action, which would explain, e.g., its (apparently nondirect) involvement in phosphorylation of downstream targets. For the sake of such studies, validated antibodies recognizing modified by PTMs ILK, and constructs coding for appropriately mutated ILK versions would be highly relevant. The issue of being by ILK a pseudokinase is addressed in the next chapter.

### Other ways of ILKs action modulation

Regulation of ILK functioning evaluated as phosphorylation of downstream targets also undergoes through well soluble mediators, including growth factors and chemokines [28, 78–82]. ILK might function here via, e.g., interaction with other molecules possessing catalytic functions or direct recruitment of kinases phosphorylating downstream targets, but somewhat less likely as a kinase.

## Molecular architecture of ILK

Initial analysis of the ILK sequence, comprised of 452 amino acids, distinguished N-terminal ankyrin repeat domain (ARD) containing four ankyrin repeats linked with a C-terminal kinase domain (KD) by a PH-like domain [43]. Further crystallography studies of the 192-amino-acid-long N-terminus of ILK in complex with the LIM1 domain of human PINCH1 revealed that the ARD domain includes five ankyrin repeats (ANK 1–5) (Figs. 2 and 3). Each consists of a pair of antiparallel α-helices separated by a short loop and packed against one another. The interior region of the superhelical spiral created with these stacked repeats forms an “ankyrin groove” that facilitates the interaction with PINCH1 [83].

### Docking of ILK to focal adhesion (FA) by interaction with other proteins

Early studies showed that deletion of ANK1 preventing PINCH from binding to ILK abolishes, in turn, the docking of ILK to FAs [4]. Going further, the fourth LIM domain of PINCH facilitates interaction with Nck-2, enabling the formation of a complex between ILK and Nck-2 and therefore create a physical link between the integrin-mediated ILK signaling pathway and growth factor-mediated or small GTPase-mediated signaling pathways [84].

Initial studies indicated that the presumable PH domain binds phosphatidylinositol-3,4,5-trisphosphate (PtdIns(3,4,5)P_3_) (PIP_3_) [78, 85]. This domain indeed shares sequence homology with other PH domain-containing proteins such as cytohesin-1 and general receptor for phosphoinositides 1 (GRP1) capable of binding PIP_3_ [78]. However, the subsequent structural analysis excluded the possibility of interaction between PIP_3_ and ILK. It is now believed that the PH-like domain is an integral part of the P loop of the KD of ILK, and between the N-terminal ILK ARD and C-terminal KD, there is a 14-residue linker domain that is either unstructured or partially structured [86]. The KD is engaged in ILKs interaction with three Parvin isoforms: α-Parvin, β-Parvin (affixin)**,** and γ-Parvin [3, 6, 87, 88] through a conservative CH2 (calponin homology) domain present in Parvin structure, which takes part in targeting of the IPP complex to FA sites [3, 7]. Recruitment of preassembled IPP to FAs is dependent on KD, which requires stabilization by binding to the chaperone Hsp90 because of its inherently unstable conformation [89]. The interaction of KD with Hsp90 seems to enhance the interaction between ILK and Parvin, stabilizing the KD’s conformation [71, 90]. All components of the IPP complex, especially ILK and PINCH are mutually dependent on each other not only in the complex formation but also in the maintenance of their protein level, which is regulated by degradation in proteasomes in the case of depletion of one of them [91]. Referring to Pichlo and Wickström, unpublished work, this degradation seems to occur independently of CHIP E3 ligase activity [11], and the exact mechanisms of IPP complex turnover are still to be clarified by further studies. In Fig. 3A, B, there are shown structures of ILK (ANK1-5):PINCH1 (10–72) and ILK (KD):α-Parvin (243–372) complexes based on the crystallographic data. Taking advantage of that data and appropriate software, we generated a structural representation of the IPP complex (Fig. 3C).

A short motif located within the KD, responsible for binding Leucine-rich (LD) sequences of ILKs molecular partners, facilitates interaction with paxillin, which takes part in the targeting of ILK to FAs [5]. That interaction is crucial for the regulation of actin cytoskeleton functions since paxillin LD motifs enable the interplay of paxillin with proteins, such as focal adhesion kinase (FAK), vinculin, Arf-GAP (GTPase-activating protein), paxillin-kinase linker (PKL), and the actin-binding protein actopaxin [5]. Apart from the LD motif, paxillin also contains the LIM domain at its C-terminus, which is crucial for its localization to FAs and facilitates interaction with cytoskeletal components, such as tyrosine–protein phosphatase nonreceptor type 12 (PTP-PEST) and tubulin [92].

One of the main functional properties of ILK relies on the end of its C-terminal domain, facilitating the binding to cytoplasmic domains of β1 [1] and β3 integrins [1, 9] (Fig. 2). It was hypothesized to play a role in the recruitment of ILK to FAs [1]. However, molecular details of this interaction still await clarification. Fukuda with colleagues showed that ILK directly binds cytoplasmic tails (CT) of integrins β1 and β3, but without revealing which amino acids are involved in that process [3]. Interestingly, a recent study implementing conservation-guided mapping has shown a previously unknown binding site for kindlin-2 on the C-lobe of the KD of ILK, which might shed light on this topic. It was further evaluated that interaction between ILK and kindlin-2 is pivotal for cell spreading and localization of both proteins at a FA site, representing a fundamental signaling axis downstream of integrins [93]. Regions of ILK binding its most important partners are shown in Fig. 2.

### ILK is most probably a pseudokinase

ILK is known to affect several downstream pathways. However, there is still substantial controversy on its exact role in those interactions. It is still under debate whether it possesses a Ser/Thr kinase activity as originally thought or remains just a scaffold protein, with a possibility of other mechanisms of involvement in signal transduction. There are contradictory reviews either determining ILK as pseudokinase [2] or those that do not yet agree with that premature judgment [94]. Although the ambiguities on catalytic capacity arising from sequence analysis seem to be supported by extensive genetic [13, 23, 24] and structural [3, 14] studies, some older in vitro analyses provide evidence for the kinase activity of ILK [6, 25, 94–96].

In the light of uncertainties surrounding the ILKs mode of action as a kinase, it has to be noted that ILKs KD binds Mg^2+^-ATP, which is known to be crucial for conventional kinase catalysis. Most of the point mutations within the ATP-binding domain were crucial for ILK’s structural integrity/stability, which is why the role of Mg^2+^-ATP was hard to assess. A recent study involving a structurally stable ILK mutant with Leucine substitution at position 207 with Tryptophan, which makes ILK to be sterically unable of ATP binding, indicated that this process is at least partially crucial in noncatalytic functions of ILK associated with F-actin bundling, such as stress fibers formation, cell spreading, and migration [97]. Other point mutation, namely K220M was found to disrupt ATP binding to ILK and cause cellular defects [14, 98]. Fukuda et al. assessed number of residues involved in ATP binding [51] (Fig. 2). Studies on recombinant KD of human ILK produced in *E. coli* showed that this domain indeed binds Mg^2+^-ATP, but it is not hydrolyzed, which is indispensable for the catalytic reaction of phosphorylation [3]. Moreover, it was proven that recombinant full-length ILK did not exhibit kinase activity towards myelin basic protein (MBP) or PKB/Akt. It turned out that the pseudokinase site conformation of KD is responsible for ILKs interaction with α-Parvin. The same study showed that partially purified endogenous ILK from chicken tissue was capable of MBP phosphorylation, but it was rather due to contamination of the chicken extract and not due to claimed ILK activity as a kinase.

Although discussing ILKs action regulation, it is crucial to acknowledge the existence of an ILK inhibitor, QLT267, also known as QLT-267 or QLT0267, which is commercially available. Several studies are employing this compound [99–104]. However, caution should be taken while using this inhibitor as it has never been unambiguously characterized in literature, whether it directly binds to ILK or is specific. To support that, results published recently suggest that QLT267 does not target solely ILK, as the results obtained upon the usage of it were diverging from the outcomes obtained upon* ILK* expression knockdown [105].

Concluding this section, there are many strong pieces of evidence supporting the notion that ILK functions solely as a scaffold protein. However, there has not been still resolved the structure of the full-length wild-type ILK of human origin produced in mammalian system. Thus, no biochemical studies have been done on such recombinant ILK in the aspect of ILKs kinase activity. We want to stress that in the following sections, we do not refer to ILK’s activity as direct phosphorylation of other proteins but as a cascade of events leading to phosphorylation of downstream targets, although in several cited here articles ILK’s action is perceived as a functional kinase.

## ILKs interactome and its cellular functions

**Fig. 4:**
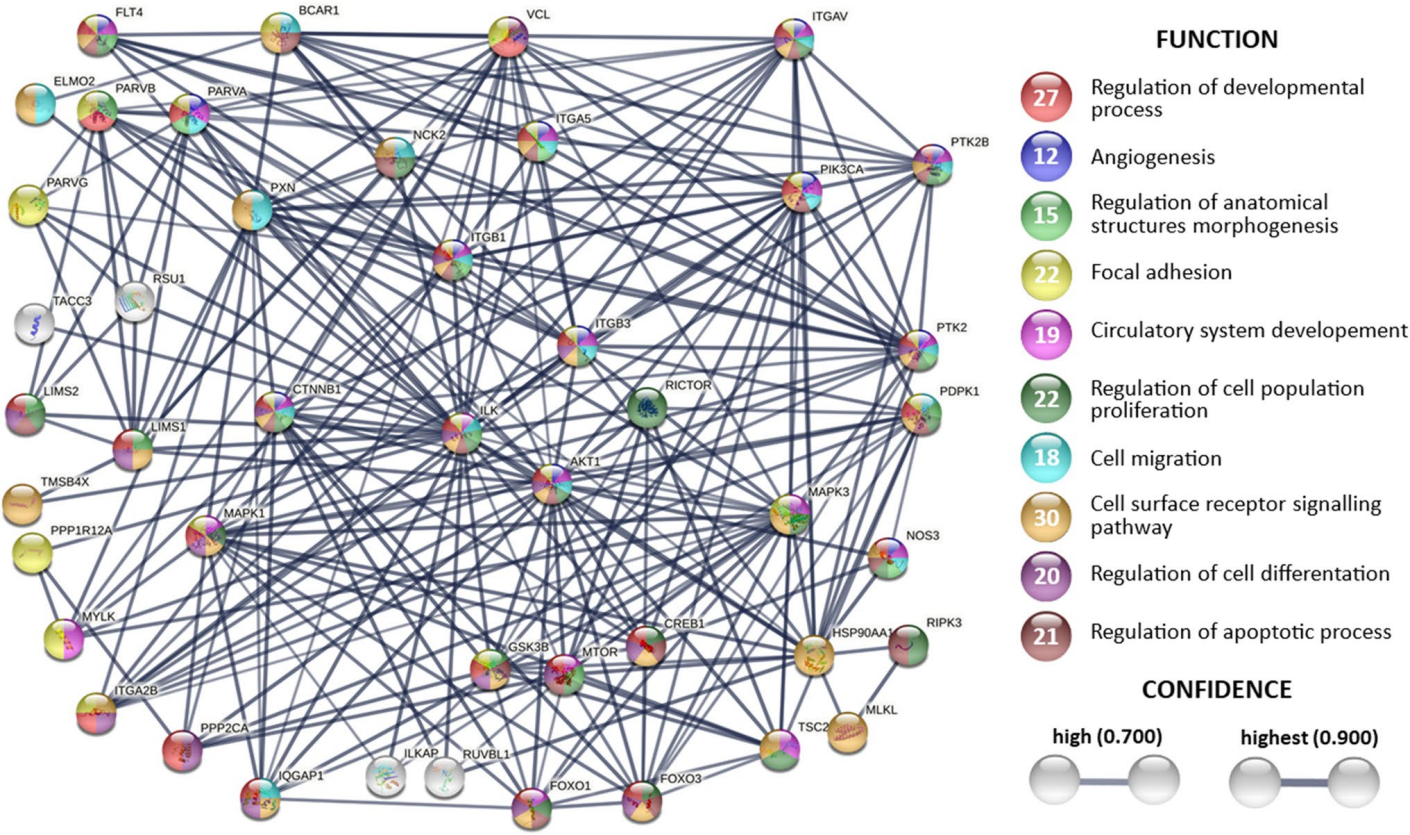
Network of the functional protein–protein interactions of ILK. The in-depth literature review supports displayed genes coding for ILKs molecular partners. The analysis was performed by STRING [Search Tool for the Retrieval of Interacting Genes/Proteins (v11.0)] provided by the STRING Consortium (Available online: https://string-db.org/) [112]. The result shows functional and physical protein associations presented in the confidence mode—the thickness of the lines indicates the strength of data support. Only records with confidence interaction mode 0.7 or higher are shown. The figure includes automatically generated connections up to 3^rd^ level and records introduced manually in line with the content of this manuscript. Textmining was excluded from interaction sources, leaving following: Experiments, Databases, Co‑expression, Neighborhood, Gene Fusion, Co‑occurrence. It is crucial to note that not all those interactions have been studied on a biochemical level. Thus, some strings might represent rather indirect interactions. Additionally, some of the automatically annotated interactors coded by the following genes: *BCAR*, *FOXO1/3*, *PDPK1*, *PIK3CA*, *PPP2CA*, *PTK2* and *TSC2* have not been studied in the context of ILK as far as we know

ILK interacts with several proteins taking part in signaling cascades involved in cell death and survival, differentiation, proliferation, migration, and many other processes in mammalian cells, projecting on respective systemic changes (Fig. 4). Amongst the main downstream targets of ILK following may be mentioned: Akt/PKB, GSK3β, β-catenin, p44/42 MAP kinases (ERK1/2), the myosin light chain (MLC) [94] as well as Merlin’s phosphatase MYPT1 (myosin phosphatase target subunit) in the Hippo pathway [106]. Akt, involved in regulating cell survival and apoptosis, is activated by phosphorylation on Threonine 308 and Serine 473. The latter occurs as a result of the activity of PI3K [25], the Rictor-mTOR complex [26], and the ILK-Rictor complex [107]. Inactivation of GSK3β through ILK-mediated phosphorylation at Serine 9 is required for regulation of cell cycle by fluctuating amount of cyclin D1 and activation of the transcription factors, such as AP-1 (activator protein 1), β-catenin/Tcf, and CREB (cAMP-response element-binding protein) [108]. Induced AP-1 upregulates the expression of genes coding for matrix metalloproteinases (MMP-9, MMP-2), thus involving ILK in pathways of invasion and metastasis [109, 110]. Furthermore, inactivated GSK3β projects onto β-catenin stabilization and subsequent accumulation, which in consequence regulates proliferation, migration, and differentiation [109]. Moreover, ILK’s action causes phosphorylation of a transcriptional co-activator of AP-1, NAC [111].

Despite the controversies on its exact function, the involvement of ILK in all of the abovementioned pathways is indisputable, and their further exploration may potentially lead to the clarification of its mechanism of action.

### Adhesion and migration

Modulation of cytoskeletal organization and processes of cell spreading and migration through ILK action includes activation of a guanine-nucleotide exchange factor (GEF) for Rac1 and Cdc42, PAK-interactive exchange factor (PIX or ARHGEF6) [33]. ILK-associated c-Src activity correlates with increased phosphorylation of cofilin at Serine in 3rd position, inhibiting its actin-severing activity and thus promoting actin polymerization [32]. Also, ILK-dependent phosphorylation of MLC on Serine 18/Threonine 19 regulates cell contraction, motility, and migration [96]. Although ILK lacks actin-binding properties, the assembly of the IPP complex triggers F-actin filament bundling, which generates contraction force and remains a mechanical signal to promote cytoskeleton reassembly and cytoskeleton-dependent cell adhesive processes such as cell shape change, cell migration, and proliferation [97]. It has been proved that ILK takes part in the modulation of actin dynamics (for review see: [11]) through regulation of small GTPases such as RhoA and Rac; however, the molecular details of these processes still awaits clarification [34–36].

Most of the mammalian cells are conditionally dependent on an interaction between integrins and ECM, and therefore disruption of those processes leads to apoptosis. Such type of programmed cell death, specifically termed anoikis [113, 114], underlies the development of normal tissues as well as prevents adherent-independent cell growth and attachment at undesired locations, which would lead to colonizing of distant organs, commonly occurring in metastatic cancer (for review see: [115]). Integrin-mediated signal transduction involving ILK is pivotal for anoikis protection. It was thought to be associated with phosphorylation of PKB/Akt on Serine 473, thus stimulating its activity [116]. Several studies highlighted the contribution of ILK and guanine-nucleotide exchange factor (GEF) to anoikis protection [117–120]. However, the role of ILK-mediated interplay with integrins in those survival pathways does not require FAK activity, suggesting that those two proteins modulate anoikis in different, parallel pathways [115, 116, 121].

### Mitosis

A quite early summary of the fundamental mitotic roles of ILK has been already included in the review by Fielding et al. [12], but further molecular details have been evaluated since then. ILK is known to be recruited to centrosomes by RUVBL1/2, where it facilitates mitotic spindle assembly through the promotion of Aurora A kinase/TACC3/ch-TOG complex formation [122]. Also, centrosome clustering is dependent on ILK through its interaction with the microtubules (MTs) regulating proteins TACC3 and ch-TOG [123, 124]. Furthermore, some studies demonstrated that ILK takes part in the regulation of MT dynamics since paclitaxel resistance and a shorter duration of mitosis have been observed in cells overexpressing ILK. From the opposite, inhibition of ILK leads to suppressed MT dynamics [125]. The vital role of ILK in the process of mitosis was supported in studies on the inhibition of ILK, which caused a G2-M-phase arrest in glioblastoma-cell lines [126, 127]. Mitotic spindle orientation, and therefore the axis of cell division, is closely dependent on the function of α-Parvin and ILK. At the same time, the latter serves as a linker between integrins and the dynactin complex. In vitro experiments on tissue-specific ILK knockout mice confirmed those phenomena, which displayed disrupted spindle orientation and cell proliferation in intestinal epithelial cells [128]. Recent evidence revealed ILKs interactor, tensin 3, to be a part of a distinct class of ECM adhesive structures that mediate attachment during mitosis, named reticular adhesions (RA). The discovery of those structures shed light on cellular adhesion during cell division since classical adhesion complexes, such as FAs, disassemble to enable mitotic rounding. The formation of RAs, mediated by integrin αvβ5, occurs during interphase, and after that they facilitate the cell–ECM attachment throughout mitotic rounding and division. Although there is no direct evidence of the ILKs involvement, the presence of the ILK interactor in those structures might indicate a new intriguing topic to be investigated. On the other hand, ILK might not be a component of RA as F-actin was not found in this adhesive structure [129].

### Endocytosis

According to early reports, the ILK sequence contains a caveolin-binding motif, and the presence of ILK was confirmed in caveolae-enriched membranes. Taking this concept further, Meyer with colleagues hypothesized that the caveolin-binding domain of ILK and the caveolin scaffolding domain of cav-1 are involved in ILK:caveolin complex formation [130]. The implication of ILK in the process of endocytosis is associated with regulation of microtubular stability, which is crucial for appropriate trafficking of vesicles with caveolin-1 to the cell surface. For that purpose, ILK recruits the scaffold protein IQGAP1 to the cell cortex, which, together with its downstream effector mDia locally stabilizes MTs and allows stable caveolae insertion into the plasma membrane [131]. Investigations of the establishment of cell–cell contacts in differentiating keratinocytes brought to light the pivotal role of ILK and Engulfment and Cell Motility 2 (ELMO2) protein in positioning E-cadherin-containing recycling endosomes [132]. The latest study also indicates the role of ILK in the endosomal recycling of N-cadherin [133]. The authors rightly noted another yet hypothetic contribution of ILK in dynamin-1 dependent clathrin-mediated endocytosis, known to be regulated through Akt/GSK3β [134], which in turn is widely known to be dependent on ILK in some way. In that view, it should be noted that many Rab proteins involved in the process of endocytosis participate in the regulation of the Akt/GSK3β signaling pathway [133, 135, 136]. Essentially, the level of ILK by itself might be also regulated through the endocytic–lysosomal pathway. The nitric oxide induction in endothelial cells resulted in decreased ILK protein stability, caused by dissociation of the complex ILK/Hsp90/endothelial NO synthase (eNOS) followed by increased ILK ubiquitylation mediated by the E3 ubiquitin ligase CHIP. The exact mechanism of ILK degradation has been positively verified through its colocalization with both lysosomal-associated membrane protein 1 (LAMP-1) and early endosome marker EEA1 (early endosome antigen 1). Also, the crucial role of dynamin in those processes has been confirmed [72].

## Subcellular distribution

Sound evidence on ILK level and localization in cells and tissues both on protein and mRNA level is based on wide immunocytochemical, immunohistochemical, and RNA-seq studies [137–140]. As a natural consequence of its function, ILK is mainly localized to cell membrane—both on the peripheral and cytoplasmic sides [6], in the FA sites and cell junctions [6, 141, 142]. In skeletal muscle, higher levels of ILK are observed predominantly at myotendinous junctions and costameres in mice and zebrafish [143, 144]. When writing about submembranous localization of ILK it would be worth evaluating whether ILK is or is not a component of reticular adhesion [129]. The presence of ILK in the cytosol is required for preassembling of the IPP complex before its recruitment to FAs [89]. Co-localization of ILK with tubulins has been observed and with centrosomal and mitotic spindle associated proteins at centrosomes [122].

Interestingly, some early reports declared its nuclear localization as well [69, 75, 145], and the process is believed to be dependent on ILKs phosphorylation [69, 75]. Still, to date, this issue has not been dealt with in-depth. Indeed, we observed such localization in our research on some melanoma cell lines (unpublished data), which corresponds with previous data reported by others on MCF-7 (epithelial-like breast cancer) [75] and A431 cells (epidermal carcinoma) [138]. Going further, it has been suggested that ILK associates with chromatin, thus acting as a gene repressor. Going further, ILK seems to play an essential role in maintaining nuclear integrity [75]. Lastly, a recent study has pointed out the presence of ILK in endothelial progenitor cell-derived exosomes [146].

What is more, the ILK’s cellular distribution pattern might be characteristic of the functional type of cells. An interesting tendency has been observed in neurons, in which the distribution of ILK varies among the cell body, being more abundant in the axonal tips than in the dendritic tips [31]. Lately, our team explored ILKs distribution in the chicken cells of peripheral nervous system, proving its localization both intracellularly and on the cell membrane of DRG neurons and Schwann cell precursors [147]. However, this observation needs to be corroborated with other methods. Those observations indicate the need for further investigations of the subcellular distribution of ILK and its importance for ILKs functioning in various types of cells and diverse cellular research models**.**

## Tissue distribution

**Fig. 5:**
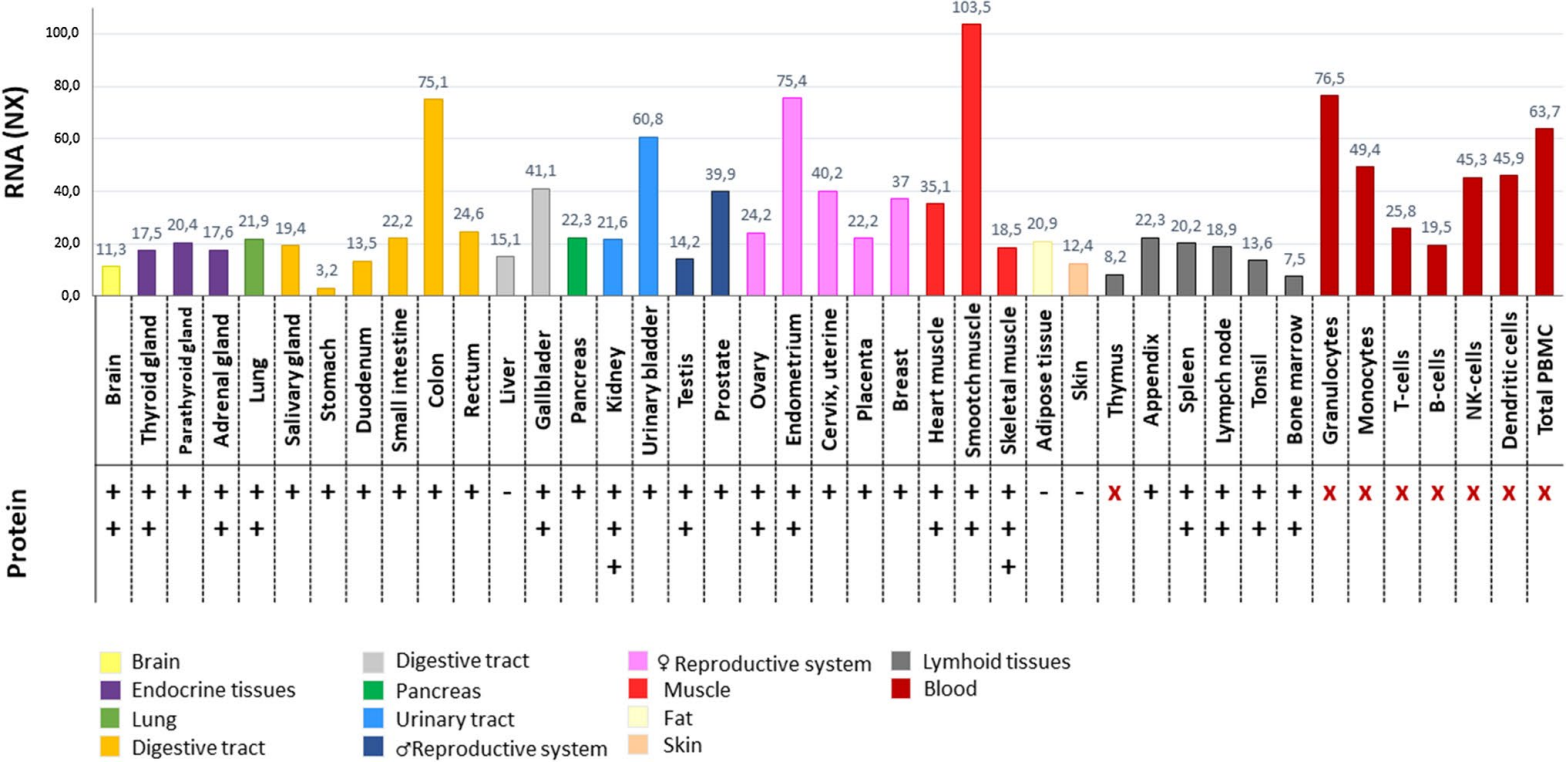
Expression of *ILK* on mRNA and protein level in human tissues. Consensus Normalized eXpression (NX) levels were created by combining the three transcriptomics datasets (HPA, GTEx, and FANTOM5) using the internal normalization pipeline. Protein level was assessed with immunofluorescent staining of cells and tissues. Relative expression level was marked as follows: +  +  + high, +  + medium, + low,—no expression, x—no data available. Color-coding is based on tissue groups. The data were collected from the Human Protein Atlas (http://www.proteinatlas.org), [138]. NK-cells—natural killer cells

No evident tissue or cell line specificity has been described for this protein. However, a significantly elevated level of *ILK* expression was detected in muscle cells, especially smooth muscle cells [6]. Various studies reported the presence of ILK in the serum of cancer patients; however, further investigation is needed to assess whether it originates from exosomes present in serum or is free-floating secreted ILK [148–151]. It is crucial to note that ILK does not possess a classical export signal peptide. Overview of the latest data on *ILK* expression in human tissues has been summarized in Fig. 5.

## Roles of ILK in physiology and disease

**Fig. 6:**
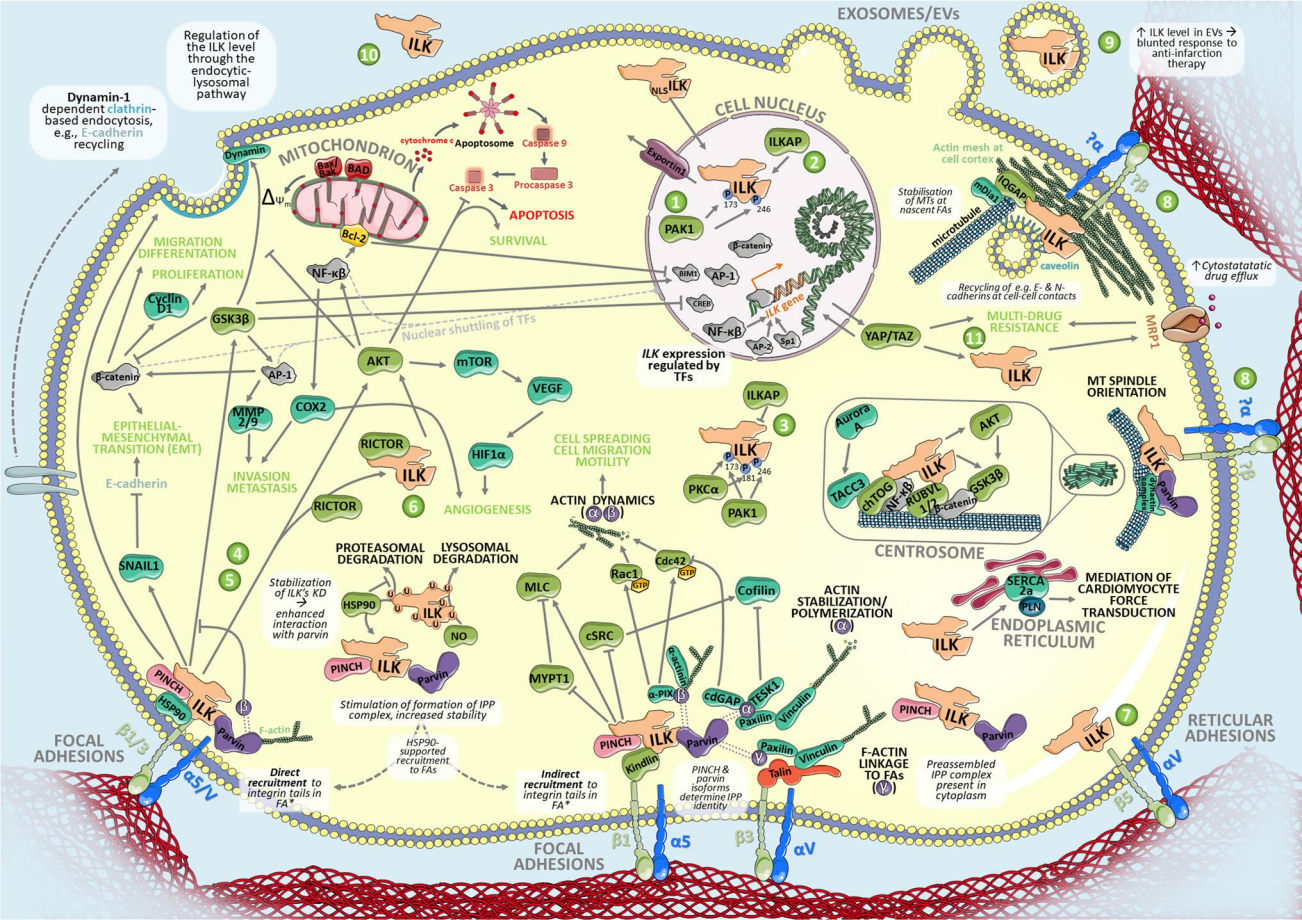
ILKs activity through signaling pathways, its interactions on a cellular level and subcellular localization. For the details, please see the text. Asterisks indicate pathways that may be initiated at a focal adhesion (FA) site regardless of the direct or indirect way of ILKs docking to FA. For the sake of simplification, the impact of FA-dependent ILK’s action on downstream effectors has been displayed as one of those. Please note that there are numbers in the figure, which refer to the issues and questions posed in Fig. 7. To highlight pathways related exclusively to particular isoforms of Parvin, α-, β-, and γ-Parvins are symbolically indicated by spheres connected to the silhouette of Parvin or shown in parentheses. The shape of ILK was modeled after the one included in the excellent review on ILK written by Widmaier and colleagues [152]. It is crucial to mention that not all of the interactions shown here might be direct, especially when it comes to phosphorylated downstrean targets of ILK. As mentioned in the text, there might be a kinase/kinases which upon binding to ILK phosphorylate, e.g., Akt or GSK3β. Nevertheless, ILK’s action impacts all proteins and pathways presented in the graphic. BAX-Bcl-2-associated protein X; BAD-Bcl-2 antagonist of cell death; Bcl2-B-cell lymphoma 2; EVs-extracellular vesicles; GTP—Guanosine-5′-triphosphate; MRP1—multidrug resistance protein 1; NLS—nuclear localization signal; NO—nitric oxide; P-phosphorylated residues of ILK; TFs—transcription factors, U-ubiquitin. Color coding: ILK’s interactors—green, downstream targets—turquoise, transcription factors—grey; in the apoptotic pathway: pro-apoptotic proteins—red, anti-apoptotic proteins—yellow; some selected proteins have been highlighted in other colors

The last two decades have witnessed a prospective growth of interest in the implications of ILK in physiological and pathological processes, which resulted in several early reviews [152–158]**.** Here we provide a summarized and updated view on those issues. Apart from the topics mentioned above, the presumed role of ILK in organismal aging and cellular senescence is worth mentioning. Both topics are covered in a recent mini-review by Olmos et al. [17]. Thus, we do not address those issues in this review. We neither planned a separate chapter about ILKs role in apoptosis as its involvement in that process is discussed in several subchapters of this section. However, to graphically summarize information included in this chapter and partly in the previous ones, we present major findings about ILK in Fig. 6.

**Fig. 7:**
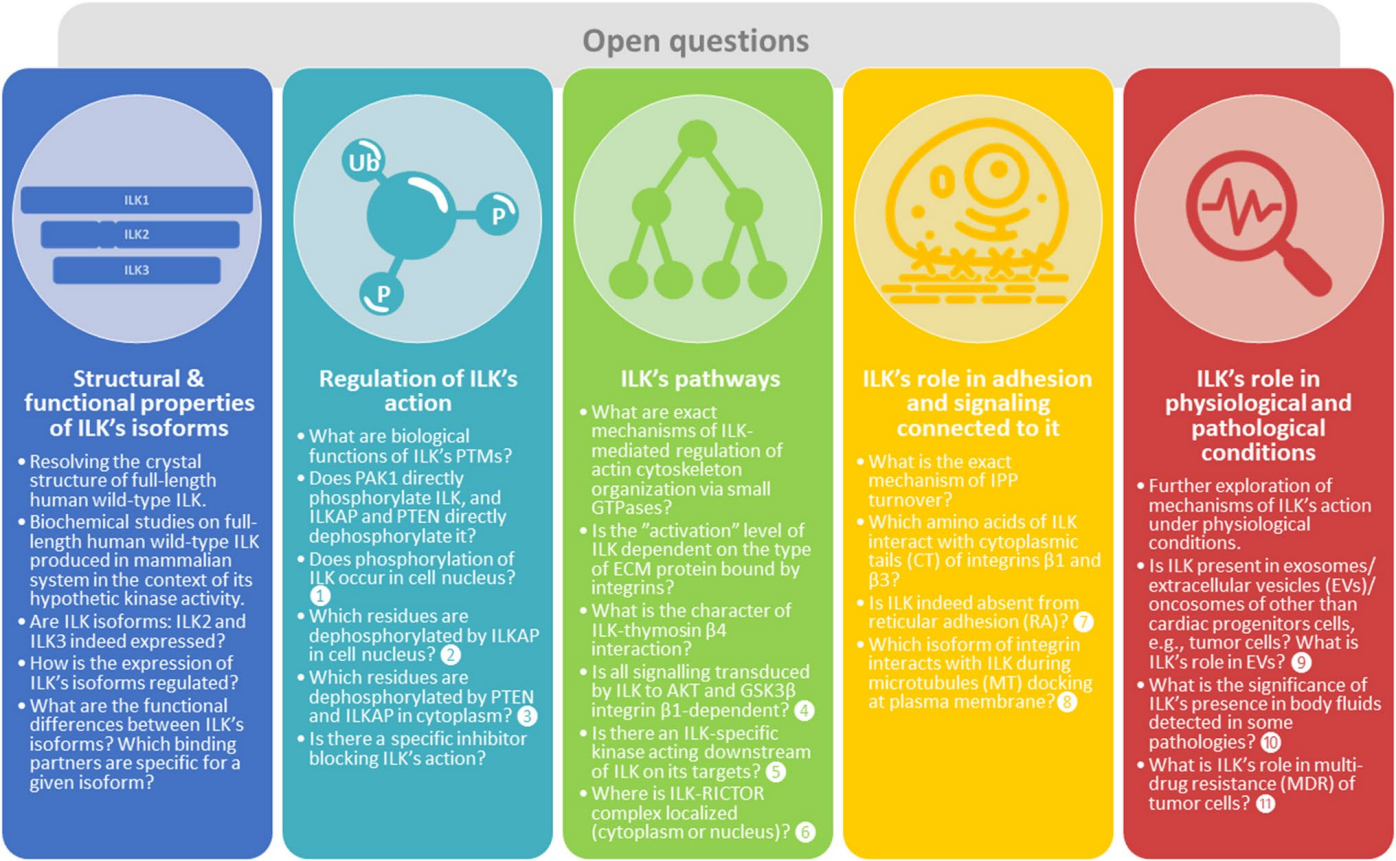
Graphical summary of the most important issues and questions which have to be addressed in future studies on ILK. The majority of the points have been elaborated in the text. Some of the questions/issues are annotated in Fig. 6. In such cases, next to them, there are numbers to be found in Fig. 6

### Cell differentiation and embryogenesis

Apart from its valid role in cell division, ILK is clearly associated with cells’ differentiation and tissue development during embryogenetic growth and adult life. Since the noncatalytical function of ILK is required for the epiblast polarization, mice devoid of *Ilk* expression die at the peri-implantation stage, unable of cavitation [13]. Mutagenesis studies on mice have shown that the presumable kinase activity of ILK is dispensable for mammalian development. Yet, an interaction between Ilk and α-Parvin emerged as critical for kidney development [14]. Mice carrying a mutation preventing Ilk from binding to α-Parvin die owing to renal agenesis, and this effect resembles the phenotype of α-Parvin-null mice [14]. Depletion of the ILK ortholog in *Xenopus laevis* impaired blastospore closure and axis elongation, with no impact on the mesodermal specification. Going further, it was concluded that XeILK is essential for morphogenetic movements during gastrulation [15]. Noteworthy, there is a strong correlation between elevated *ILK* expression and enhanced differentiation in normal gastrointestinal, neural, bone marrow, renal tissue, and more differentiated areas of malignant tumors [159]. During human endometrial decidualization, ILK was demonstrated as pivotal for the morphologic transformation of endometrial stromal cells (ESCs) through the organization of the actin cytoskeleton. That was confirmed by *ILK* knockout, with the characteristic features of abrogated polymerization and organization of actin fibers, which reverted the cells to their undecidualized morphology [16]. Moreover, ILK remains a vital component of keratinocyte differentiation programs triggered by increased extracellular Ca^2+^, concentration projecting on epidermal integrity, and the establishment of barrier properties of the epidermis [160]. Signal transduction orchestrated by ILK is also necessary for TGF-β1 triggered dermal fibroblast transition to myofibroblast (Dermal Myofibroblast Differentiation, DMD) [161].

### Epithelial to mesenchymal transition

Epithelial to mesenchymal transition underlies the invasive and migratory abilities of cells, therefore participating in tumor progression and metastasis and fibrosis. ILK has been demonstrated to promote lung cancer cell migration and invasion through the induction of the EMT process [162]. Moreover, ILK emerged as a therapeutic target in neoplastic transformation and EMT induced by expression of v-ets erythroblastosis virus E26 oncogene-like (avian) gene and prostate cancer [163] as well as in the high glucose-induced EMT of renal tubular cell [164]. An increase in ILKs level correlates with a decrease of E-cadherin (known as epithelial cell marker) and an increase of vimentin and N-cadherin (mesenchymal markers) amounts. This transition, called a “cadherin switch,” is a significant hallmark of EMT [110, 165]. Essentially, ILK has been proven to orchestrate endosomal recycling of N-cadherin and E-cadherin [132, 133].

Going further, ILK is crucial for TGF-β1-induced EMT [28, 166] through a Snail and Slug mechanism [39]. Significantly, an increase in ILK level lowers the amount of E-cadherin through upregulation of its repressor Snail, which promotes EMT leading to invasion and metastasis [39, 110]. On the contrary, siRNA-mediated silencing of *ILK* expression significantly reduced the nuclear presence of proteins like Snail, Twist, Zeb, and β-catenin and reversed the “cadherin switch” [165]. More recent evidence from studies on patients and chemoresistant colon cancers further evaluated the correlation of *ILK* expression and the level of a wide range of EMT markers [167]. Targeting this pathway seems to be a promising approach for the treatment of fibrotic kidney [166], lung [168], prostate [169], or bladder cancer [170]. Interaction between ILK and Rictor seems to be crucial in TGF-β-mediated EMT, since its prevention, either by silencing of *ILK* expression or by applying the ILK inhibitor, blocks this process and even partially reverses the mesenchymal phenotype in breast cancer cell lines [106]. More recent evidence highlights NF-κB as another molecular player modulated by ILK in EMT promotion [27, 28]. Finally, ILK plays a crucial role in Twist-induced EMT, as a part of the Twist-integrin β1-FAK/ILK pathway [171]. Expression of *ITGB1*, gene encoding integrin β1, is positively regulated by Twist on the transcriptional level. Followingly, integrin β1 activates both FAK and ILK signaling axes through phosphorylating FAK and upregulating *ILK* expression [171, 172], which are both necessary for activating essential for EMT MAPK/ERK, PI3K/Akt, and WNT signaling [171].

### Nervous system

Along with its downstream targets, such as Akt and GSK3β, ILK was implicated in the development and function of neurons, especially dendritic morphogenesis and neurite outgrowth [29–31]. For instance, downregulation of *ILK*s expression leads to inhibited axonal growth, causing either length reduction or elimination of the axon, whereas “hyperactivation” of ILK promotes the formation of multiple axons [31]. Another approach in the contribution of ILK in axon formation might be related to inactivation of the MYPT1 leading to accumulation of MLC phosphorylation or phosphorylation of MLC [96, 173]. The latter was confirmed to be implicated in multiple axons formation [174]. ILK is involved in cytoskeleton and adhesion dynamics in oligodendrocytes, which are associated with its role in growth cone maturation through MTs regulation. Moreover, cells with ILK depletion displayed decreased process length and myelin production capacity [175]. The latest studies conducted in our lab proved ILKs involvement in the chicken peripheral nervous system development dependent on laminin-1 via modulating axonal outgrowth, sensory neuron morphology, and Schwann cell precursors number [147]. A study on embryonic hippocampal neurons suggested the role of ILK in neuroprotection through the Akt-dependent integrin survival signaling pathway [176]. A more recent paper evaluated the ILK-mediated survival pathway stimulated by insulin, projecting on the regulation of survival kinases such as Akt and GSK3β and to inhibition of caspase-3 activity. Those observations were in line with *ILK* expression knockdown and ILK inhibition experiments, which resulted in decreased insulin-mediated neuroprotection [177]. In vivo experiments on a rat model of Fetal Alcohol Spectrum Disorder (FASD) revealed a close association of impaired ILK pathway and synaptic plasticity, leading to FASD-related memory alterations [178]. Following the observation of significantly decreased ILK protein levels in streptozotocin (STZ) mice, a model for Alzheimer’s Disease (AD), it has been also suggested that ILK may participate in the pathogenesis of AD [179]. Taking this concept further, the latest evidence has shown reversion of the hippocampus-dependent neurogenesis and memory deficits in APP/PS1 mice, another AD model, via the Akt–GSK3β pathway triggered by increasing the level of ILK. What is more, the application of the serotonin reuptake inhibitor improved the impaired hippocampal neurogenesis and memory by enhancing activation of the ILK–Akt–GSK3β axis, suggesting ILK as a promising therapeutic target for AD prevention and treatment [180]. On the other hand, targeted deletion of the *Ilk* gene in the central nervous system in the mice model caused cortical–lamination defects typical for Cobblestone lissencephaly [181] as well as loss of nerve growth factor (NGF) signaling [182]. Interestingly, depletion of ILK had no direct effect on cell survival. However, the reduced proliferation rates of granule-cell precursors have been observed in both studies [181, 182]. The growth-suppressive function of ILK was also concluded from studies on the neural stem and progenitor cells, describing a signaling cascade controlled by IPP complex through RSU-1 (Ras suppressor protein 1) mediated regulation of JNK activity projecting on proliferation both in vitro and in vivo [183]. Those and many other observations largely resemble the phenotype of central nervous system-restricted integrin-β1-knockout mice [184], which supports the claim that ILK is the leading player of integrin-β1-dependent processes in the brain [158].

### Dermal and musculoskeletal system

ILK is involved in various cellular processes in keratinocytes, such as proliferation, adhesion, spreading, and migration [69, 185]. Depletion of ILK in the skin leads to skin blistering and impaired hair follicle development [69, 185]. It has been suggested that ILK modulates epidermal regeneration following injury [186], which corresponds well with delayed wound closure in skin observed in ILK-deficient mice [187]. Integrin-linked kinase is indispensable for developing epidermis and hair follicles, because ILK-deficient melanoblast displayed in vivo abnormalities in forming long pseudopods and thus resulted in impaired migration. Moreover, their proliferation was affected. Those effects were observed also in mature melanoblasts, accompanied by disruptions in cell responses to the extracellular matrix substrates collagen I and laminin 332 [188]. Adverse effects of the ILK inactivation are partially restored by the Rac1 activity, which points out the potential importance of integrin-linked kinase-Rac1 nexus in melanocytic cell establishment, dendricity, and functions in the skin [188].

During skeletal development, ILK-induced pathways are crucial for the proliferation and differentiation of chondrocytes within the growth plate, while depletion of ILK in chondrocytes causes dwarfism and chondrodysplasia [189, 190]. An in vivo study has shown that the ILK-nascent polypeptide-associated complex and Coregulator alpha cascade (αNAC) reduce the pace of osteoblast maturation and downregulates its mineralization [191]. Also, mechanosensing and signaling in vertebrate skeletal muscle depend on ILK proper functioning but not on its presumed kinase activity [144]. ILK has been found to participate in Ca^2+^-independent smooth muscle contraction via inhibition of myosin light chain phosphatase and thus increased myosin phosphorylation [96]. ILK is implicated in negative regulation of skeletal muscle proliferation through muscle cell enhancement factor 2C influencing phosphorylation activity and muscle creatine kinase (MCK) mRNA level. Such regulation occurs independently of PI3K [192]. It is known that phosphorylation of Akt, a downstream target of ILK, plays a substantial role in the regeneration of skeletal muscle.

What is more, the integrin β1-ILK complex is recognized as an essential component of IGF-1R/insulin receptor substrate signal transduction to Akt during mechanical stress in skeletal muscle. Mice with a skeletal muscle-restricted knockout of *Ilk* suffer from a mild progressive muscular dystrophy, mainly within the area of myotendinous junctions, co-occurring with a detachment of basement membranes from the sarcolemma and accumulation of ECM in myotendinous junctions [143]. Postel et al. investigated the role of ILK in muscles, proving that it is recruited to the myotendinous junctions, and the presence of laminin and itgα7 is necessary for this process. Although ILK occurred to be crucial for mechanical stability in skeletal muscles in zebrafish, its participation in dystrophin/dystroglycan adhesion complex is not necessary in that process. Suprisingly, ILK did not seem to be required for formation of the adhesion complex, but still it improves strengthening the adhesion of the muscle fibre with the ECM, which is dependent on the presence of the pseudokinase domain [144]. It has been pointed out, that diet-induced muscle insulin resistance shall relate to ILK through the interaction with collagen-binding integrin which participates in the expansion of ECM. Further evidence suggested a cause–effect relationship between *Ilk* expression in muscle and impairment of insulin signaling and insulin perfusion through capillaries [193].

### Kidney

Formation and phosphorylation of the PINCH1-integrin-linked kinase-α-Parvin (IPP) complex was proved to be crucial for controlling podocyte adhesion, morphology, and survival. Not only cellular levels of PINCH1, ILK, and α-Parvin, cytoplasmic components of cell–ECM adhesion complexes, were elevated throughout podocyte differentiation but an increased amount of the PINCH1–ILK–α-Parvin complex was detected in differentiated podocytes as well [194]. Essentially, ILK is required to maintain the glomerular filtration barrier and might be involved in the progression of the glomerular dysfunction. It is associated with ILKs pivotal antiapoptotic role in human mesangial cells through a mechanism involving integrin β1/ILK/Akt-dependent NF-κB activation causing overexpression of an antiapoptotic protein, Bcl-xL, which in turn counteracts ILK/GSK3β-dependent expression of proapoptotic factor, Bim [195]. The ILK-regulated NF-κB pathway is implicated in renal inflammation processes and thus might be considered as a possible therapeutic target for inflammatory renal diseases [196]. ILK is involved in proteinuria and has also been implicated in podocyte cell–matrix interaction and the distribution of nephrin and α-actinin [197, 198]. Specific renal *Ilk* ablation in mice caused progressive focal segmental glomerulosclerosis, leading to death in terminal renal failure [198]. The phenotype of *Ilk* knockout mice resembled alterations observed in chronic renal diseases, such as the impaired urine concentration or tubulointerstitial fibrosis of the kidney. It therefore may constitute a nephrogenic diabetes insipidus model (NDI) [199]. Going further, ILK has been suggested as a therapeutic target in NDIs since it regulates the level and localization of tubular water channel aquaporin-2 (AQP2) which directly affects renal reabsorption [199]. Another study on mice demonstrated that Ilk controls branching morphogenesis by regulating the expression of dual-specificity phosphatase 8, which inhibits p38 MAPK activity resulting in a decrease of branching morphogenesis [200]. A recent study has shown that the expression of *Ilk* in renal stroma is essential for multiple aspects of renal development. Mice with kidney-specific *Ilk* depletion exhibited a considerable decrease in ureteric bud branching and defected collecting duct formation. Those mice displayed renal vasculature mispatterning and impaired glomerular vascular differentiation, which was caused by the disruption of several key signaling pathways required for kidney morphogenesis, including PI3K/Akt and MAPK/ERK [201]. Going further, mice with specific depletion of *Ilk* in collecting duct (CD) principal cells (PCs) demonstrate interstitial fibrosis and inflammation associated with activating the canonical TGF-β signaling cascade. Ilk-deficient CD PCs died by necroptosis through activation of the Receptor Interacting Serine/Threonine Kinase 3 (RIPK3) and mixed lineage kinase domain like pseudokinase (MLKL) pathways, highlighting the critical involvement of Ilk in this process [202].

### Heart

The integrin β1/ILK/β-Parvin network has been proposed as a mechanosensor for stretching forces in the heart [203]. Deleting *Ilk* distorts adhesion signaling through the integrin β1/FAK complex resulting in the disaggregation of cardiomyocytes [21]. Not only do mutations in *ILK* contribute to cardiac hypertrophy and contractility, but ILK is a novel cardiotropic factor promoting the transformation of human fetal heart cells to a cardiomyogenic fate as well [18]. It also has a protective role against cardiomyopathy and heart failure in mammals as activated by thymosin β4, actin sequestrating protein, and it stimulates the function of cardiomyocytes and healing after infarction [204–206]. Curiously, we have shown recently that thymosin β4 locates to FAs in melanoma cells, where ILK is found [207] pointing at ILKs and thymosin β4 potential interaction in melanoma cells. Tantos with colleagues found out that thymosin β4 interacts directly with both the 4th and 5th LIM domains (amino acids 189–325) of human PINCH1 and the N-terminal Ankyrin repeat domain (amino acids 1–160) of human ILK [208]. Those interactions are weak and transient and reflect the nature of thymosin β4, i.e., being an intrinsically disordered protein, which explains the moonlighting character of thymosin β4. Intrinsically disordered proteins might interact with other proteins without changing their conformation. That phenomenon is called “binding without induced folding,” and though such interactions are weak, they are specific and essential for cellular processes [209].

Point mutations in *ILK* and *LAMA4* coding for laminin α4 have been suggested as a direct cause of dilated cardiomyopathy (DCM) in humans through simultaneous defects in endothelial cells and cardiomyocytes [22]. It was further investigated on rats that upregulation of integrin-linked kinase ameliorates the severity of DCM in a rat model, improving cardiac function, and decreasing mortality [210]. What is more, ILK acts as a mechanosensory element in the human heart, mediating cardiomyocyte force transduction through regulation of the vital calcium regulatory protein sarcoplasmic/endoplasmic reticulum Ca^2+^ATPase isoform 2a (SERCA-2a) and phosphorylation of phospholamban (PLN), which provides additional support for the link of ILK with DCM and highlights its potential role as the therapeutic target [20]. In general, increasing the level of ILK has a cardioprotective role [211]. The role of ILK in the reparative properties of endothelial progenitor cells (EPCs) and their exosomes on the myocardial repair was concluded from the study on mouse model mimicking systemic inflammation condition (interleukin-10 gene knockout). The high amount of ILK in exosomes caused a blunted therapeutic effect, while reducing ILKs level in those extracellular vesicles recovered inflammatory response, indicating ILK as a target protein for improving progenitor cell exosomes-based cardiac therapies [146]. A particularly interesting recent study employing rats’ hearts as an ischemia–reperfusion (I/R)-induced arrhythmia ex vivo model showed that ILKs action prevented those rats from arrhythmia, potentially through the inhibition of connexin remodeling via Akt activation [212].

### Vascular system

Stimulated by vascular endothelial growth factor (VEGF) ILK has been found to upregulate VEGF-mediated endothelial cell migration, capillary formation in vitro, and angiogenesis in vivo [213]. ILK is essential for postnatal vasculogenesis, supporting the recruitment of endothelial progenitor cells to ischemic tissue [214]. A study on mice has shown that Ilk participates in eNOS regulation, preventing it from uncoupling and, therefore, may be regarded as a therapeutic target for preventing endothelial dysfunction related to shear stress-induced vascular diseases [215]. While ILK is vital in regulating vasomotor tone, its decreased level has been observed in atherosclerosis. Data collected from studies on a mouse model of vascular remodeling (carotid artery ligation and human atherosclerotic artery samples) indicate a correlation between increased levels of inducible nitric oxide and decreased ILK levels. Those effects are dependent on enhanced degradation of ILK through endocytosis, triggered by an overload of inducible NO. Further inhibition of that pathway may represent a therapeutic target for atherosclerotic disease [72]. A recent RNA sequencing study has provided evidence on the crucial role of dysregulated cell adhesion and ILK signaling in pathogenesis of arrhythmogenic cardiomyopathy (ACM). That mechanism seems to be characteristic for ACM caused by disruptions in the *FLNC* gene encoding filamin C, a major cardiac structural protein, differing from already described pathological mechanisms underlying classic arrhythmogenic right ventricular cardiomyopathy caused by desmosomal genes mutations [216]. ILK also regulates endothelial proliferation, migration, and tube formation in the retina, and therefore may be further investigated for therapeutic purposes in ocular neovascularization [217]. Ilk-deprived mice had increased vascular content as well as elevated activity of soluble Guanylyl Cyclase (sGC) and Protein Kinase G (PKG), which in turn led to intensified vasodilatory response to NO donors [218]. Integrin-linked kinase is responsible for Ca^2+^-independent diphosphorylation of MLC20 [219], and negatively regulates Rho/ Rho-associated protein kinase (ROCK)–mediated signaling, leading to vascular smooth muscle cell (SMC) contraction, which in turn contributes to the control of vascular wall formation [36]. In the vessel wall, ILK is crucial for maintaining SMCs in a stationary phenotype but also mediates the response to injury. Abrasion of the vessel triggers a decrease in *ILK* expression resulting in SMC migration and proliferation that establishes a thickened neointima. Subsequent fibronectin deposition at the luminal edge of the vessel is associated with an increase in *ILK* expression, which promotes adhesion of SMCs, contributing to the arrest of cell migration and proliferation at this location [220]. Finally, ILK was found to mechanically regulate vascular endothelial growth factor receptor 3 (VEGFR3) signaling, impeding its interaction with integrin β1, ensuring the proper development of lymphatic vessels in vitro and in vivo [221].

## The role of ILK in cancer

One of the most explored and reviewed topics associated with ILK covers a widely studied role in tumor development, prevention, and treatment. Such an important role of ILK in tumorigenesis stems from the abovementioned implications in cell differentiation, mitosis, apoptosis, angiogenesis, EMT, and migration. Although that pivotal issue should not be overlooked in this paper, a significant part of it was already covered by others in reviews on roles of integrin-linked kinase in tumor signaling [158] and its perspectives as a therapeutic target [40, 153, 155, 157, 158, 222] or referring to specific types of cancers: hormonal [223], breast [224] or rhabdomyosarcoma [225].

In general, most of the currently available evidence suggests a pro-oncogenic function of ILK in tumorigenesis. *ILK* is frequently overexpressed in a broad spectrum of human tumors and has been connected with unfavorable prognosis in patients’ survival and treatment [148, 162, 226–236]. Much work on the therapeutic potential of genetic or pharmacological inhibition of ILK has been carried out, demonstrating significant downregulation of various oncogenic signaling pathways and thus suppression of tumor development and progression in several cancer types upon ILK’s “deactivation” [100, 101, 126, 237–244]. Curiously, the elevated ILK level in serum might be considered as a marker of various cancers, such as malignant pleural mesothelioma [148], non-small cell lung cancer [149], esophageal squamous cell carcinoma [150], and ovarian carcinoma [151]. Cancer patients had strikingly higher ILK levels in serum compared to a barely detectable level in healthy volunteers. The level of ILK was also negatively correlated with chemotherapy efficiency [150] and postoperative survival [149], while positively correlated with the progression of malignancy [148, 151] 

### ILK and multidrug resistance of tumor cells

In the latest advance, an increasing number of studies have highlighted a link between ILK-related pathways and multidrug resistance (MDR), especially to platinum drugs. The elevated level of ILK promoted the proliferation of human glioma cells, leading to escape from apoptosis, and lowered sensitivity to temozolomide via decreasing the activity of caspase-3. Corroborating those results it was shown that RNA silencing of *ILK* expression increased the sensitivity of the lung cancer cells to cisplatin, enhancing its apoptotic action [245]. Interestingly, ILKAP has been suggested as a regulatory hub of ovarian cancer cell susceptibility to platinum drugs [246]. Coleman with colleagues further investigated the molecular and biological effects of targeting ILK in cisplatin-resistant ovarian cancer and identified multiple target genes involved in cell growth, apoptosis, invasion, and metastasis, including those encoding noncoding RNAs. Researchers also observed reduced cisplatin-resistant cell growth and invasion ability and increased apoptotic response after siRNA-mediated silencing of *ILK* expression in ovarian cancer cells [247]. The most recent study showed that treatment with a small molecule inhibitor of ILK, QLT0267, may reduce acquired resistance to 5-fluorouracil of human colon cancer cells [167].

A growing body of literature emphasizes the multidimensional role of ILK in the MDR phenomena. It has been found that ILK mediates activation of YAP/TAZ, a co-transcriptional activator involved in the development of drug resistance, and thereby promotes tolerance to doxorubicin of breast cancer cells. What is more, cell resistance of the cells varied depending on the stiffness of the matrix, suggesting that MDR is affected as a result of the transduction of the mechanical signal to the interior of the cell through ILK-mediated YAP activation [248]. Increased levels of ILK protein have been as well associated with gemcitabine resistance of A549 cells because silencing of the *ILK* expression partially reversed that drug resistance. One of the possible reasons for that phenomenon is explained with evidence: ILK regulates MRP1, a multidrug resistance protein 1, critical for the efflux of gemcitabine, and that ILK’s action is possibly dependent on epithelial–mesenchymal transition [249]. In chronic myeloid leukemia (CML) therapy with tyrosine kinase inhibitors (TKIs), genetic and pharmacological inhibition of ILK has been found to sensitize resistant leukemic stem cells (LSCs). It was noted that only TKI-refractory LSCs from patients, but not normal hematopoietic stem cells were eliminated following ILK inhibition [48].

## Summary, conclusions, and open questions

Based on studied for this review literature, we identified several gaps in knowledge about ILK. Thus, we decided to pose some important questions that future studies should address. Those questions are shown in Fig. 7.

While many researchers unambiguously classify ILK as *bona fide* pseudokinase, it raises a fundamental question: what is ILK’s precise role and mechanism of action in various cellular processes without kinase activity? Still, the issue that leaves no doubt is that ILK is essential for described in this work signaling events, which was already supported by multiple evidence from in vitro and in vivo experiments.

Because of relatively low costs and simple procedures, bacteria remain the most common expression system employed in studies on recombinant proteins [250]. However, some researchers express doubts on the reliability of that system, as it may be imprecise due to the loss in the post-translational modifications required for biological activity or proper protein folding. In the light of all controversies surrounding ILKs activity, structural studies of wild-type ILK expressed in mammalian cells could provide conclusive evidence. Going further, once a structurally stable ILK mutant devoid of Mg^2+^-ATP binding properties has been obtained [97], it could be a valuable tool in the further investigation on noncatalytic signal transduction or even shed light on controversies in ILKs kinase activity. Alternatively, finding a kinase or kinases which would act downstream from ILK on such ILKs targets as Akt or GSK3β would be a finding bringing together seemingly contradictory data on ILKs mode of action. Interaction of ILK with such hypothetical kinase/kinases might be weak and transient, which could explain why it has not been discovered yet. That kind of interaction could be similar to that between thymosin β4 and ILK or PINCH1.

Despite growing high-throughput evidence on the presence of diverse PTMs of ILK, the investigation of their functions is neglected in the current literature. Fourteen residues of ILK are known to be ubiquitylated, and it is the most common post-transcriptional modification of ILK. On the functional level, only recently, a crucial role of those modifications has been confirmed in diverse pathways of degradation of ILK. However, it is still necessary to narrow down our knowledge to precise sites of those modifications on ILK, which probably differ in each pathway. Essentially, the function of acetylation and sumoylation of ILK remains unsolved.

Moreover, among twelve phosphorylation sites, the function of only four of them, namely of Serine 246, Threonine 173, Threonine 181, and Serine 343, have been addressed in the literature but still not in-depth. Two of them are probably crucial in the regulation of nuclear shuttling and subcellular localization of ILK. Still, this issue has not been dealt in-depth and the last investigations in that topic have been published in the previous decade. Although the role of Threonine 173 and Serine 246 phosphorylation in nuclear shuttling seems to be confirmed in human cells, some concerns may arise from mice studies. Interestingly, mouse sequence of ILK overlaps with the human ILK in approximately 98%. At the same time, both of those residues are conserved and have been confirmed by high-throughput evidence to be phosphorylated in mice [49, 50], yet still, no nuclear localization of ILK has been reported in murine cells [138].

Among the most interesting yet still unexplored areas of ILK is the issue of its diverse isoforms. Based on current data, it might be assumed that structural differences between them may be associated with differential subcellular localization as the 192 N-terminal residues crucial for the nuclear localization [113, 116] are truncated in ILK2 and utterly absent in ILK3. While ILK1 and ILK3 have the conserved pseudokinase fragment, ILK2 lacks an ATP binding fragment but at this place, contains a 28aa unique fragment of unknown function. Today’s proteomic data on the ILK1 and theoretical knowledge on ILK2 and ILK3 let us presume their diverse functional characteristics.

Although embryonic lethality of *Ilk* knockout model organisms initially impeded comprehensive in vivo experiments [13, 15, 23, 24], the development of tissue-specific ILK knockouts [128] or experiments involving ILK-specific molecular inhibitors [106, 167] turned out to be a promising solution for this issue. Establishing whether QLT267 is indeed a specific ILK inhibitor is important, as the number of studies employing this compound is growing. Moreover, obtaining new inhibitors of ILK is desirable. For instance, an inhibitor targeting the interaction interface of ILK:Parvin complex could be highly appreciated because IPP complex formation seems to be essential for the well-being of tumor cells. On the other hand, such compound should be precisely delivered to tumor tissue as ILK and IPP are important for normal cells' proper functioning.

It should be noted that the majority of the research on the cellular cascades involving ILK has been performed on transformed or tumor cells, which might have its limitations in the extrapolation of these data on the ILK activity and function in nontransformed cells or organisms. Thus, more research on normal cells is needed.

Finally, ILK has been widely suggested as a potential therapeutic target not only in human malignancies [40, 153, 155, 157, 158, 222], but also in fibrosis of kidney [166], lung [168], prostate [169], or bladder cancer [170]; Alzheimer disease [180]; inflammatory renal diseases [196]; nephrogenic diabetes insipidus (NDI) [199] or cardiovascular diseases [20, 72, 146]. Henceforward, the general issue for further studies is to evaluate if—and how—either genetic or pharmacological regulation of ILK might be successfully implemented in therapies of human diseases. The next intriguing aspect of being explored is ILK’s presence and role in the extracellular space.

In conclusion, though we already know a lot about ILKs functions in both physiological and pathological conditions, there is still a wide range of topics that have to be addressed (Fig. 7). Altogether, that makes ILK an intriguing topic to study.

## Data Availability

The authors confirm that the data supporting the findings of this study are available within the article.

## Abbreviations

AD: Alzheimer’s disease
Akt: Protein kinase B (PKB)
AP-1: Activator protein 1
ARD: Ankyrin repeat domain
CHIP: C terminus of HSC70-interacting protein
CH2: Calponin homology
DCM: Dilated cardiomyopathy
EMT: Epithelial to mesenchymal transition
eNOS: Endothelial nitric oxide synthase
ERK: Extracellular signal-regulated kinase (p44/42 MAPK)
FA: Focal adhesion
FAK: Focal adhesion kinase
FASD: Fetal Alcohol Spectrum Disorder
GEF: Guanine-nucleotide exchange factor
GSK3β: Glycogen synthase kinase 3-beta
Hsp90: Heat-shock protein 90
ILK: Integrin-linked kinase
ILKAP: Integrin-linked kinase-associated phosphatase
IPP: ILK–PINCH–Parvin complex
KD: Kinase domain
LD: Leucine-rich domain
MAPK: Mitogen-activated protein kinase
MLC: Myosin light chain
MMP: Matrix metalloproteinase
mTOR: Mammalian target of rapamycin
MTs: Microtubules
MYPT1: Myosin phosphatase target subunit 1
NF-κB: Nuclear factor-kappa B
PAK1: P21-activated kinase 1
PIP_3_: Phosphatidylinositol (*3*,*4*,*5*)-trisphosphate
PI3K: Phosphoinositide 3-kinase
PINCH: Particularly interesting cys-his-rich protein
PTMs: Post-translational modifications
RA: Reticular adhesion
Cdc42: Cell division control protein 42 homolog
SMC: Smooth muscle cell
VEGF: Vascular endothelial growth factor
TGF-β1: (Transforming Growth Factor β1)
UTR: Untranslated region
